# Development of fluorescence-labeled antibody for immune checkpoint inhibitor using engineered probiotics

**DOI:** 10.1186/s13568-023-01509-y

**Published:** 2023-01-12

**Authors:** Fu Namai, Shunsuke Sumiya, Natsumi Nomura, Takashi Sato, Takeshi Shimosato

**Affiliations:** Department of Biomolecular Innovation, Institute for Biomedical Sciences, Shinshu University, 8304 Minamiminowa, Kamiina, Nagano, 399-4598 Japan

**Keywords:** gmLAB, Next-generation probiotics, PD-L1, scFv, Tumor microenvironment

## Abstract

Here, we developed a genetically modified lactic acid bacteria (gmLAB) that produces green fluorescent protein (GFP)-conjugating, anti-programmed death-ligand 1 (PD-L1) single-chain variable fragments (scFv) for use as an anti-cancer device that targets immune checkpoint molecules. Since PD-L1 plays a key role as an immune checkpoint molecule in the tumor microenvironment, inhibition and detection of PD-L1 are important in cancer research. The anti-PD-L1 scFv was designed based on atezolizumab, a humanized IgG1 monoclonal antibody, and integrated into a lactococcal GFP gene expression vector. Gene expression from the constructed gmLAB was confirmed by western blotting and GFP fluorescence. The ability of GFP-conjugating anti-PD-L1 scFv against the target antigen, PD-L1 protein, was shown using an enzyme-linked immunosorbent assay. Finally, the ability to recognize PD-L1-expressing tumor-cell lines was confirmed using flow cytometry and fluorescence microscopy. Our results suggest that the gmLAB could be applied to in vivo imaging in cancer as an affordable diagnostic/treatment tool.

**Graphical Abstract:**

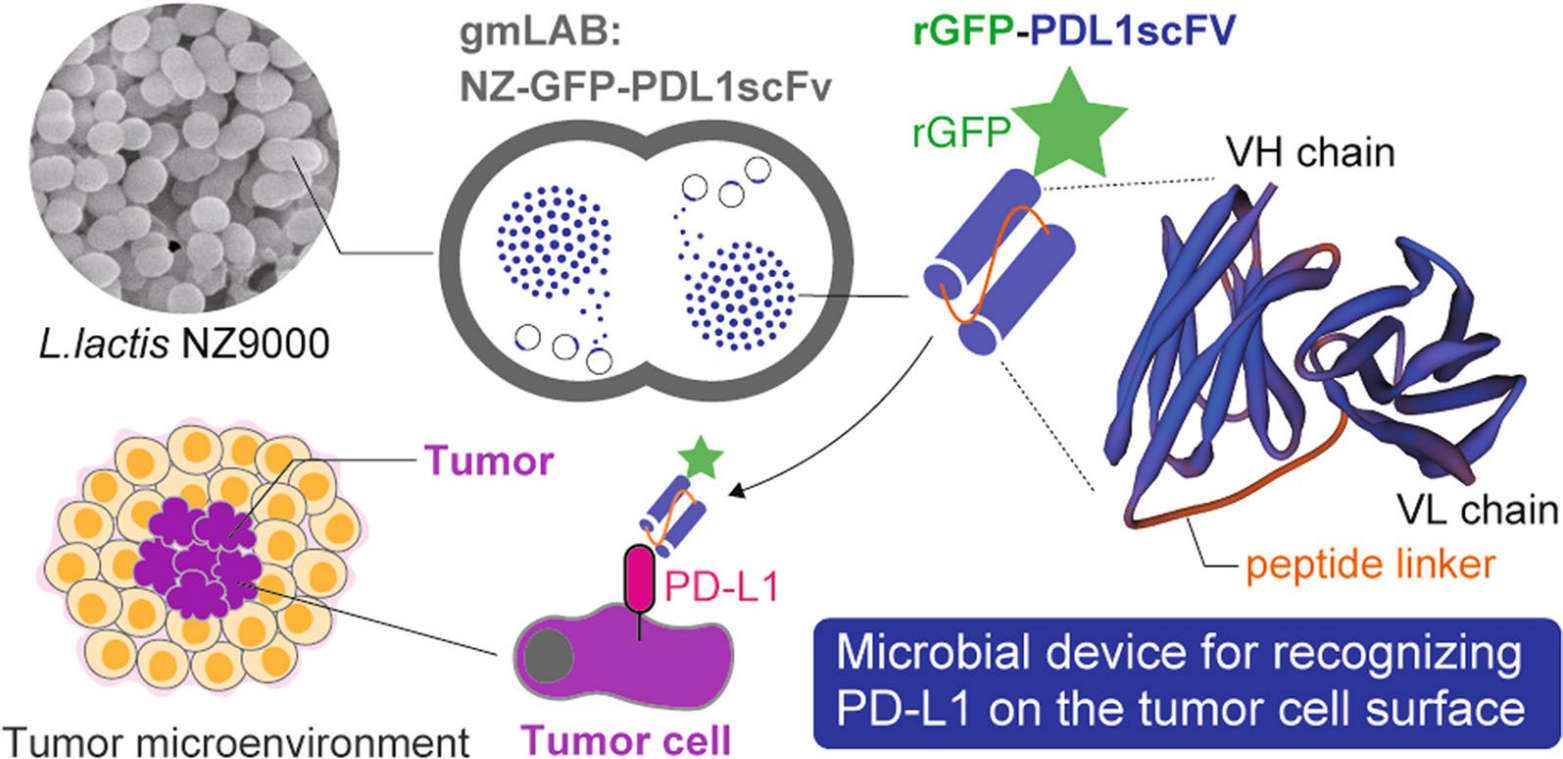

## Key points

- Successfully constructed a microbial device based on the gmLAB-producing GFP-labeled anti-PD-L1 scFv.
- Recombinant GFP-PDL1scFv exhibits immunoreactivity against the PD-L1 protein on the tumor cell surface.
- The gmLAB has the potential to be used as an affordable diagnostic tool for treating cancer.

## Introduction

Lactic acid bacteria (LAB) are among the most extensively studied human symbiotic gut bacteria and fermenters of livestock products and have been classified by the US Food and Drug Administration (FDA) as generally being safe for use in the food sciences (Hill et al. 2017). Although considerable research has been conducted on improving quality of life by utilizing the functionality of LAB, their application as biopharmaceuticals has been anticipated in recent years. In particular, the use of genetically modified LAB (gmLAB) has gained attention in the prevention and alleviation of mucosal disorders (Shigemori and Shimosato 2017). gmLAB can be used to produce a variety of recombinant proteins by transforming cells with gene expression vectors. gmLAB are ingested and remain viable in the intestinal tract, where they can produce recombinant proteins in situ; as a result, they have been studied for use as an intestinal drug delivery system for recombinant proteins (Cano-Garrido et al. 2015). In addition, a recent study showed that intranasal administration of gmLAB can also deliver proteins to the respiratory mucosa, thereby contributing to disease relief (Yumoto et al. 2020). In this context, gmLAB capable of producing beneficial or therapeutic proteins are classified as next-generation probiotics (NGPs) or microbial therapeutics and are expected to be exploited further in the future (Jimenez et al. 2019; O’Toole et al. 2017).

In this study, we constructed a strain of gmLAB that produces a single chain variable fragment (scFv) fused with a green fluorescent protein (GFP) that is capable of recognizing programmed death ligand 1 (PD-L1), and that can be used as a tool for the treatment, alleviation, and diagnosis of cancer. PD-L1, known as an immune checkpoint molecule, is an important target in cancer therapy (Dermani et al. 2019). Therefore, anti-PD-L1 antibodies, which are immune checkpoint inhibitors (ICIs) that can inhibit the interaction between PD-L1 and its receptor, programmed death 1 (PD-1), can be applied clinically as powerful antitumor agents (Jiang et al. 2019b). Antibody drugs are expensive to manufacture due to their sophisticated structure, but scFv, which contains an antigen recognition site for an antibody connected by a flexible peptide linker, can be produced in a prokaryotic expression system and thus can be procured at a low cost (Arbabi-Ghahroudi et al. 2005; Samaranayake et al. 2009). In addition, the presence or absence of PD-L1-positive cells in the tumor microenvironment can have a significant impact on the selection of an appropriate treatment strategy (Jiang et al. 2019a), making the simple detection of PD-L1-positive cells a critical issue. Therefore, we considered that constructing a gmLAB strain capable of producing GFP-fused anti-PD-L1 scFv (GFP-PDL1scFv) could be an attractive alternative antibody drug and diagnostic/treatment tool. This study aimed to design an scFv based on the anti-PD-L1 antibody, construct a gmLAB strain that produces GFP-PDL1scFv, and verify the PD-L1 protein recognition ability of GFP-PDL1scFv.

## Materials and methods

### Bacterial strains, growth conditions, and plasmid

*Lactococcus* (*L*.) *lactis* subsp. *cremoris* NZ9000 (NZ9000) was purchased from MoBiTec GmbH (Gottingen, Germany). NZ9000 is a derivative of *L*. *lactis* subsp. *cremoris* MG1363 in which the *pepN* gene has been replaced with the constituent genes of the NICE system, i.e., *nisR* and *nisK* (Ruyter et al. 1996). NZ9000 was cultured in M17 broth (BD Difco^™^, Becton, Dickinson and Co., MD, USA) containing 0.5% glucose (GM17) at 30 °C without shaking. Constructed gmLAB were cultured using GM17 supplemented with 10 µg/mL chloramphenicol (GM17cm). *Escherichia* (*E*.) *coli* MC1061 was purchased from MoBiTec GmbH and cultured using Luria-Bertani (LB) broth (Invitrogen Corp., CA, USA) containing 25 µg/mL chloramphenicol (LBcm) at 37 °C with intense shaking.

**Fig. 1:**
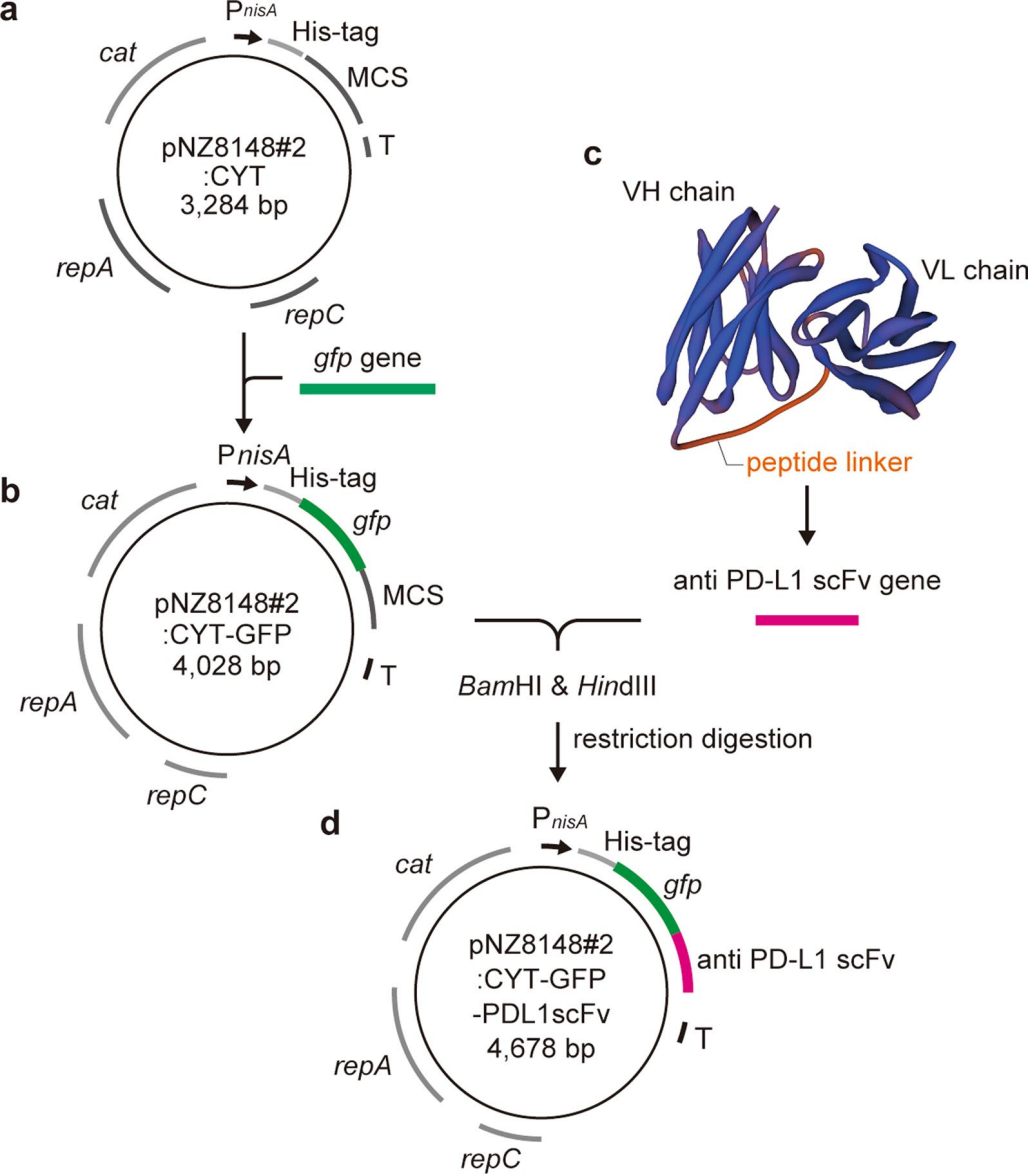
Scheme of plasmid construction. **a** The lactococcal gene expression vector, pNZ8148#2:CYT, derived from commercially available pNZ8148. **b** The green fluorescent protein (GFP) gene was integrated into pNZ8148#2:CYTo construct the lactococcal GFP expression vector, pNZ8148#2:CYT-GFP. **c** Predicted 3D model of PDL1scFv designed using SWISS-MODEL. The locations of the VH chain, VL chain, and peptide linker (EGKSSGSGSESKS) are shown. VH chain; variable region of the heavy chain, VL chain; variable region of the light chain. **d** The PDL1scFv gene was integrated into pNZ8148#2:CYT-GFP by restriction digestion using *Bam*HI and *Hin*dIII to construct GFP-conjugated PDL1scFv expression vector, pNZ8148#2:CYT-GFP-PDL1scFv. P_*nisA*_: nisin-inducible promoter, *His-tag* six-histidine tag, *MCS* multiple cloning site, *T* terminator, *repC and repA* origins of replication, *Cat* chloramphenicol acetyltransferase, *GFP* green fluorescent protein

The NICE-system plasmids, pNZ8148#2:CYT and pNZ8148#2:CYT-GFP, were constructed based on the commercially available pNZ8148 (MoBiTec GmbH), as described in Shigemori et al., and were used as the gene expression vectors (Fig. 1a, b) (Shigemori et al. 2012, 2017b).

### Design of the scFv and construction of the gene expression vector

PDL1scFv was designed by connecting the amino acid sequences of the variable region of atezolizumab using the flexible peptide linker EGKSSGSGSESKS. The three-dimensional (3D) structure of the designed PDL1scFv was predicted using SWISS-MODEL, an automated protein homology-modeling server (Schwede et al. 2003). The designed amino acid sequences were then converted to nucleotide sequences by Eurofins Genomics (Tokyo, Japan), based on *L. lactis* subsp. *cremoris* MG1363 codon usage. In addition, restriction enzyme recognition sites, *Bam*HI and *Hin*dIII, were inserted on each side of the scFv sequence. The resulting gene was subcloned into pEX-K4J2 by Eurofins Genomics (Tokyo, Japan). General molecular cloning techniques were performed using modifications of previously described methods (Namai et al. 2018a). Briefly, the gene segment was excised using *Bam*HI and *Hin*dIII and cloned into the multi-cloning site of pNZ8148#2:CYT-GFP. The resulting GFP-conjugated PDL1scFv (GFP-PDL1scFv) expression vector (designated pNZ8148#2:CYT-GFP-PDL1scFv) was sequenced by Eurofins Genomics (Tokyo, Japan) to confirm the absence of mutations and/or deletions.

### Construction of gmLAB for GFP-PDL1scFv gene expression

pNZ8148#2:CYT-GFP-PDL1scFv (DDBJ accession number: LC739557) was introduced into NZ9000 to construct a gmLAB strain (designated as NZ-GFP-PDL1scFv) by electroporation (Namai et al. 2018a). Simultaneously, pNZ8148#2:CYT and pNZ8148#2:CYT-GFP were also introduced into NZ9000 to generate the vector control gmLAB (designated as NZ-VC and NZ-GFP). The constructed gmLAB were then cultured to induce gene expression (Namai et al. 2018b). Briefly, the pre-incubated gmLAB were inoculated into GM17cm (final concentration 5%), and nisin, a gene expression inducer, was added when the optical density at 600 nm (OD_600_) reached 0.4 (1-1.5 h). Cells were harvested at 3 h after the addition of nisin by centrifugation (4 °C, 8,000×*g*, 5 min) and washed with ice-cold Tris-buffered saline (TBS: 50 mM Tris-HCl, 140 mM sodium chloride, pH 8.0) or phosphate-buffered saline (PBS; 137 mM sodium chloride, 2.7 mM potassium chloride, 10 mM disodium hydrogen phosphate, 1.76 mM potassium dihydrogen phosphate, pH 7.4). Then, the cell pellets were crushed using a bead beater (µ-12; TITEC, Saitama, Japan), and the soluble fraction was obtained by centrifugation (4 °C, 20,000×*g*, 15 min). An equal volume of 2× sample buffer (Wako, Osaka, Japan) was added to the soluble fraction and boiled at 95 °C for 5 min to prepare the sample for western blotting (WB) (Ishida et al. 2020; Namai et al. 2018a).

### Confocal laser scanning microscopy

Freshly prepared TBS-washed bacterial pellets were suspended in 400 µL of TBS, and 10 µL was placed on a microscope slide and observed under a confocal laser scanning microscope (FluoView FV1000, Olympus, Tokyo, Japan) using an oil immersion objective lens (×60).

### Immunoreactivity assay of GFP-PDL1scFv

Recombinant gene expression was induced, and the cell pellet was crushed to obtain a soluble fraction containing recombinant protein, as described above. The total protein concentration of the soluble fraction was measured using a BCA Protein Assay Kit (Thermo Fisher Scientific, MA, USA) according to the manufacturer’s instructions, and the solution was adjusted to 5 mg/mL. The immunoreactivity of the recombinant GFP-PDL1scFv (rGFP-PDL1scFv) was examined using an enzyme-linked immunosorbent assay (ELISA), as described previously (Namai et al. 2020a, b; Shigemori et al. 2017a).

### Purification of rGFP-PDL1scFv

Recombinant gene expression was induced by adding nisin (final: 1.25 ng/mL) to 1 L culture, as described above. The cell pellet was then collected by centrifugation (4 °C, 8,000×*g*, 5 min), washed using MilliQ water, and frozen at −80 °C. The frozen pellets were pulverized into a fine powder in liquid nitrogen using a Cryo Press disruptor (Microtec Co., Chiba, Japan), followed by adding 10 mL binding buffer (20 mM imidazole, 20 mM Na_3_PO_4_, 0.5 M NaCl, pH 7.4). The soluble fraction was collected (4 °C, 12,000×g, 15 min), and DNA was removed using a Nucleic Acid Removal Kit (ProFoldin, Hudson, MA, USA) as per the manufacturer’s instructions. The resulting fraction was filtered using a DISMIC-25AS filter (pore size 0.45 μm, Toyo Roshi, Tokyo, Japan). The filtrate was loaded onto a HisTrap HP column (1 mL, GE Healthcare) equilibrated with binding buffer, and the column was washed with five column volumes (CV) of binding buffer. The column-absorbed proteins were then eluted with a linear gradient of 0–500 mM imidazole over 40 CVs at 1 mL/min using a fast protein liquid chromatography system (AKTA pure 25, GE Healthcare). The collected fractions (soluble fraction; cell, wash, flow-through, and eluate; F1-8) were analyzed by WB with CBB staining following SDS-PAGE, as described above. The eluted fractions were then dialyzed against PBS, and the His-tagged protein concentration in the dialyzed sample was measured using a His-Tag ELISA Detection Kit (GenScript, Piscataway, NJ, USA).

### Culture conditions for Raw264.7 cells

The mouse macrophage cell line, Raw264.7 cells (ATCC, Manassas, VA, USA), was maintained in complete Dulbecco’s Modified Eagle’s medium (DMEM; with 10% fetal bovine serum [GE Healthcare], penicillin [100 U/mL; Nacalai Tesque, Kyoto, Japan], and streptomycin [100 µg/mL; Nacalai Tesque]) at 37 °C in 5% CO_2_ and passaged once every three days.

### Flow cytometry of Raw264.7 cells

Raw264.7 cells were seeded on a 24-well plate at 2.0 × 10^6^ cells/well and cultured at 37 °C for 2 h. After removing the supernatant, a complete DMEM containing 0 or 10 µg/mL of lipopolysaccharide (LPS; InvivoGen, San Diego, CA, USA) was added. After 4 h of incubation at 37 °C, cells were collected by centrifugation (4 °C, 500×*g*, 5 min) and washed with PBS containing 1% fetal bovine serum. Cells were then stained using 1/100 dilution of PE anti-mouse CD274 (B7-H1, PD-L1) Antibody (BioLegend, San Diego, CA, USA) or purified rGFP-PDL1scFv (500 ng/mL) at RT for 1 h. After washing, cells were analyzed using a Cell Sorter SH800 (SONY, Tokyo, Japan), and cell populations were identified using FlowJo software (v10.5.3; BD Biosciences, San Jose, NJ, USA).

### Fluorescence observation of Raw264.7 cells

Raw264.7 cells were seeded on a 24-well plate at 2.0 × 10^6^ cells/well and cultured at 37 °C for 2 h. After removing the supernatant, a complete DMEM containing 0 or 1 µg/mL of LPS was added. After 24 h incubation at 37 °C, the supernatant was removed, and cells were fixed using 200 µL of 10% formalin neutral buffer solution for 10 min. Then, cells were washed twice with PBS containing 0.05% Tween 20 (Nacalai) (PBS-T) and stained using 200 µL of 1/100 dilution of PE anti-mouse CD274 (B7-H1, PD-L1) antibody or purified rGFP-PDL1scFv (500 ng/mL) for 1 h. Cells were washed twice using PBS-T and mounted using DAPI-Fluoromount-G (Southern Biotech, Birmingham, AL, USA). The resulting slides were observed under a BZ-X800 microscope (Keyence, Osaka, Japan).

### Statistical analysis

GraphPad Prism software (version 8, GraphPad, San Diego, CA, USA) was employed for statistical analysis, and significance was accepted at p < 0.05. For flow cytometry analysis, the data were analyzed using unpaired t-tests.

## Results

### Construction of GFP-conjugated PD-L1scFv expression vector

PDL1scFv amino acid sequences were designed based on the sequences of the anti-PD-L1 antibody, atezolizumab (KEGG Drug: D10773). A flexible peptide linker, EGKSSGSGSESKS, connected variable regions of the heavy and light chains. To confirm the 3D structure, the structure of PDL1scFv was predicted using SWISS-MODEL. The results suggested that the VH chain and VL chain, which have independent structures, were connected by the peptide linker (Fig. 1c). The resulting amino acid sequences were converted to DNA sequences based on *L*. *lactis* subsp. *cremoris* MG1363 codon usage by Eurofins Genomics (Tokyo, Japan). The restriction enzyme recognition sites, *Bam*HI and *Hin*dIII, were added to both sides of the DNA sequences and then subcloned into pEX-K4J1. PDL1scFv sequences and pNZ8148#2:CYT-GFP (Fig. 1b) derived from pN8148#2:CYT (Fig. 1a) were excised by restriction digestion to construct the GFP-conjugated PDL1scFv expression vector, pNZ8148#2:CYT-GFP-PDL1scFv (Fig. 1d).

### Construction of GFP-PDL1scFv-producing gmLAB

**Fig. 2:**
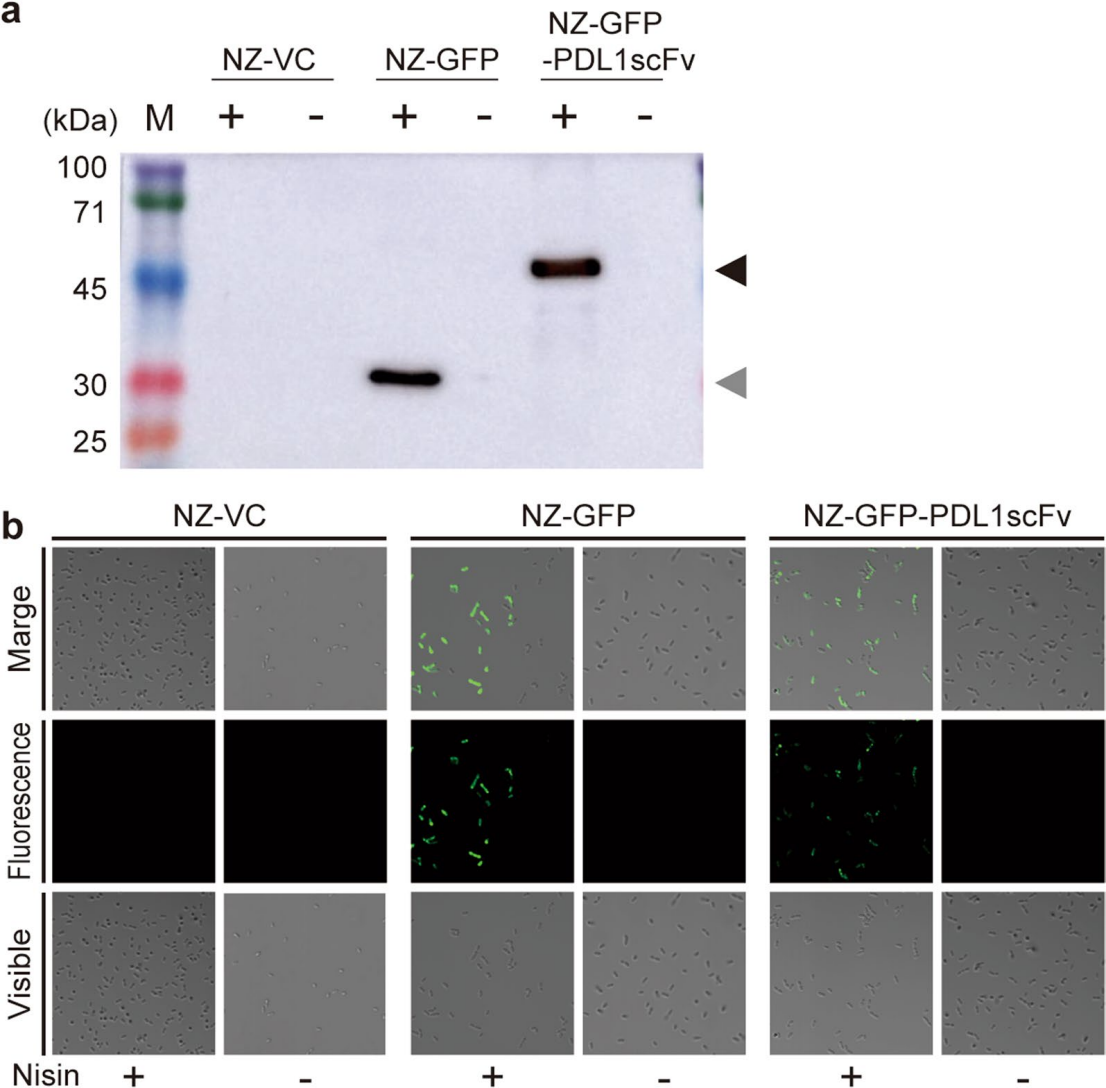
Gene expression and fluorescence analysis. NZ-VC, NZ-GFP, and NZ-GFP-PDL1scFv were cultured with/without nisin, and gene expression and fluorescence were analyzed. **a** The cell extracts were subjected to WB using an anti-His  tag antibody to detect the expression of rGFP-PDL1scFv by the gmLAB. The black arrow indicates the band corresponding to rGFP-PDL1scFv (59.0 kDa). The gray arrow indicates the band corresponding to rGFP (31.0 kDa). M: molecular mass marker (kDa). **b** Fluorescence analyses of gmLAB. Each gmLAB was observed by confocal laser scanning microscopy using an oil immersion objective lens (×60) under visible and fluorescent light, and merged images were generated. −/+: absence/presence of nisin stimulation

pNZ8148#2:CYT-GFP-PDL1scFv was introduced into NZ9000 by electroporation to construct the gmLAB strain, NZ-GFP-PDL1scFv. pNZ8148#2:CYT and pNZ8148#2:CYT-GFP were also introduced into NZ9000 to construct the vector control gmLAB strains, NZ-VC, and NZ-GFP, respectively. Each gmLAB strain was cultured with/without nisin at 30 °C for 3 h, and the cell extracts were subjected to WB. As a result, when NZ-GFP-PDL1scFv and NZ-GFP were incubated with nisin, bands corresponding to either rGFP-PDL1scFv (59.0 kDa) or recombinant GFP (31.0 kDa) were detected, respectively (Fig. 2a). In contrast, no bands were detected in the NZ-VC, NZ-GFP-PDL1scFv, and NZ-GFP samples without nisin stimulation (Fig. 2a). Next, each gmLAB strain was cultured with or without nisin. The washed cells were observed under a confocal laser scanning microscope. GFP fluorescence was observed in the NZ-GFP and NZ-GFP-PDL1scFv samples incubated with nisin. However, no fluorescence was detected in the NZ-VC, non-stimulated NZ-GFP, and non-stimulated NZ-GFP-PDL1scFv samples (Fig. 2b).

### Immunoreactivity assay of NZ-GFP-PDL1scFv

**Fig. 3:**
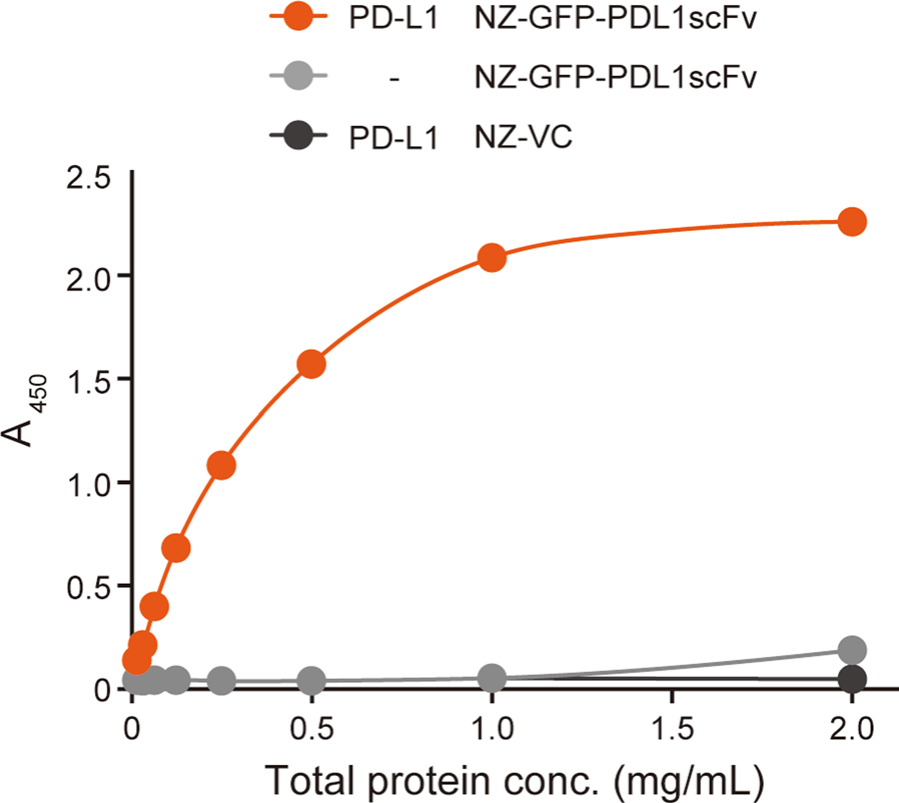
Immunoreactivity assay of NZ-GFP-PDL1scFv against PD-L1. A binding assay using an anti-His tag antibody was performed to verify the immunoreactivity of rGFP-PDL1scFv. Serially diluted cellular extracts of each gmLAB were added to wells immobilized (orange and black line) or not immobilized (gray line) with PD-L1 protein. The bound scFv was detected using an anti-His tag antibody, and the absorbance at 450 nm was  measured. The data are representative of two independent experiments. Orange line: PD-L1+, NZ-GFP-PDL1scFv, Gray line: PD-L1-, NZ-GFP-PDL1scFv, Black line: PD-L1+, NZ-VC

The antigen recognition ability of rGFP-PDL1scFv was investigated by ELISA. The target protein, PD-L1, was immobilized on a multi-well plate, and a cell extract of the nisin-stimulated NZ-GFP-PDL1scFv containing rGFP-PDL1scFv was added. The bound rGFP-PDL1scFv was detected using an anti-His-tag antibody. As a result, the absorbance at 450 nm increased with an increase in the concentration of total protein in the NZ-GFP-PDL1scFv cell extract when added to PD-L1-immobilized wells (Fig. 3). In contrast, the absorbance remained at baseline levels when the NZ-GFP-PDL1scFv cell extracts were added to wells that did not contain immobilized PD-L1 or when NZ-VC cell extracts were added to the PD-L1 immobilized well (Fig. 3). These results suggested that rGFP-PDL1scFv produced by NZ-GFP-PDL1scFv exhibits immunoreactivity against the PD-L1 protein.

### Purification of rGFP-PDL1scFv from gmLAB

**Fig. 4:**
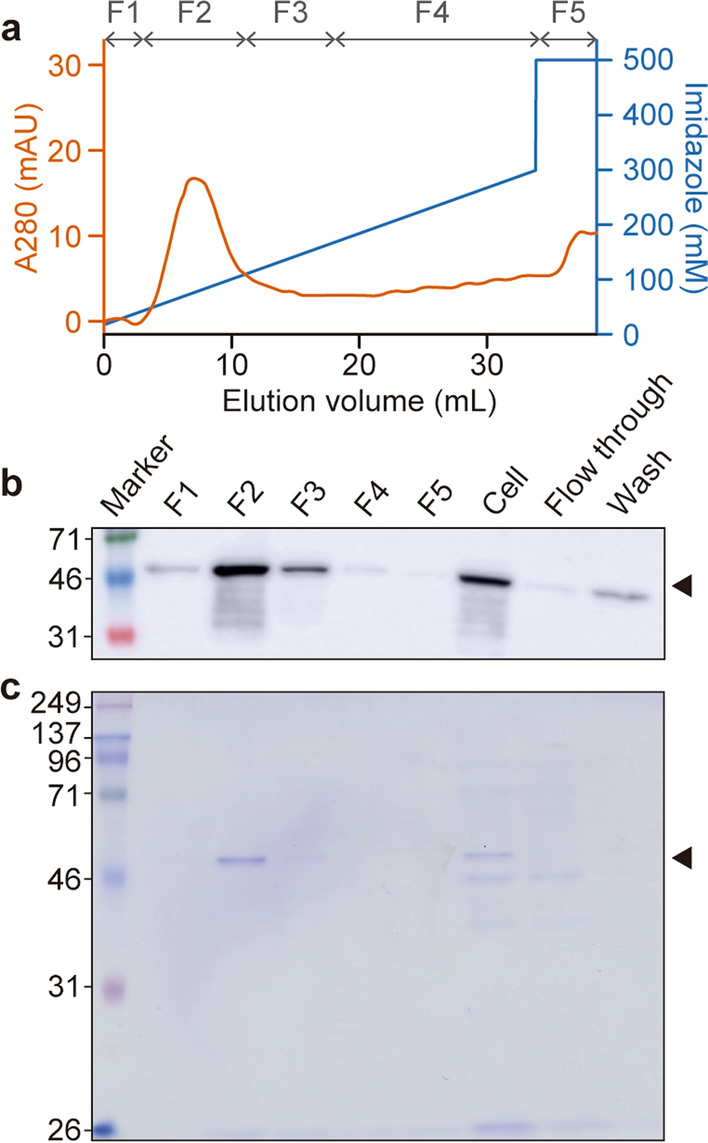
Purification of rGFP-PDL1scFv. **a** Chromatogram of protein elution. Protein adsorbed on a His-Trap column was eluted by adding imidazole. The eluted protein was collected as five fractions (F1–F5) based on A_280_. **b** The presence of rGFP-PDL1scFv in each fraction was analyzed by WB using an anti-His-tag antibody. **c** Results of SDS-PAGE (CBB staining) analysis. The black arrow indicates the band corresponding to the size of rGFP-PDL1scFv (59.0 kDa)

rGFP-PDL1scFv was purified from the NZ-GFP-PDL1scFv cell extract. A chromatogram of the absorbance at 280 nm (A_280_) is shown in Fig. 4a. Based on the absorbance values, the purified solution was separated into five fractions (F1–F5), and sample purity was assessed by WB (Fig. 4b) or by SDS-PAGE followed by staining with CBB (Fig. 4c). Bands corresponding to rGFP-PDL1scFv (59.0 kDa) and highly purified rGFP-PDL1scFv were confirmed, particularly in fraction F2. Therefore, in subsequent experiments, F2 was used as purified rGFP-PDL1scFv. The ELISA results indicated that F2 contained 2.19 µg/mL (600 µL) of rGFP-PDL1scFv.

### Cell surface antigen recognition ability of rGFP-PDL1scFv

**Fig. 5:**
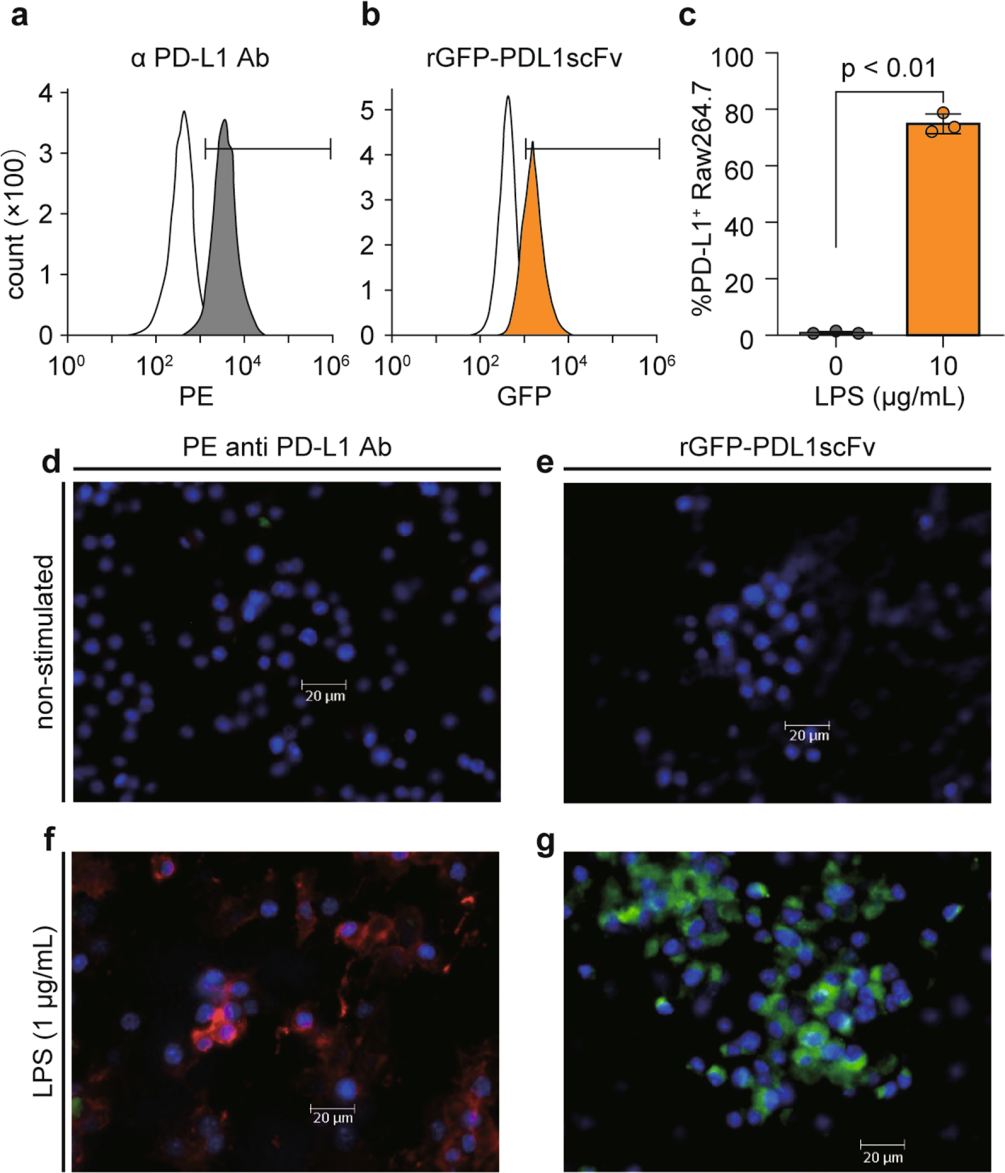
Cell surface antigen recognition ability of rGFP-PDL1scFv. Raw264.7 cells (2.0 × 10^6^ cells/well) were stimulated with lipopolysaccharide (LPS) (1 or 10 µg/mL) and stained using PE anti-mouse PD-L1 antibody or purified rGFP-PDL1scFv. Then fluorescence was detected by flow cytometry (**a-c**) and fluorescence microscopy (**d-g**). Representative flow cytometry histograms of staining with PE anti-mouse CD274 (B7-H1, PD-L1)  antibody (**a**) or rGFP-PDL1scFv (**b**) are shown, respectively. Each white histogram indicates non-stimulated Raw264.7 cells. Gray histograms indicate LPS-stimulated Raw264.7 cells, and orange histograms indicate LPS-stimulated Raw264.7 cells. Black bars indicate the gated region used to calculate PD-L1-positive cells. **c** The graph shows the measurement results for the  gated positive cell ratio in the stimulated or non-stimulated Raw264.7 cells stained with rGFP-PDL1scFv. Data are the mean ± SD (n = 3), and each dot on the plot represents one experiment. Representative fluorescence microscopy images of Raw264.7 cells stimulated with LPS (**f**, **g)** or without LPS (**d**, **e)** and stained with DAPI and PE anti-mouse CD274 (B7-H1, PD-L1) antibody (**d, f**) or rGFP-PDL1scFv (**e, g)** are shown (blue: DAPI, red: PE, green: GFP). Scale bar = 20 μm

Next, we verified whether the rGFP-PDL1scFv produced by the gmLAB recognized PD-L1 expressed on the cell surface of Raw264.7 cells. Since Raw264.7 cells express PD-L1 on the cell surface when stimulated with LPS (Xiao et al. 2020), the cells were stimulated with LPS (0, 1 or 10 µg/mL) and stained with commercially available PE anti-PD-L1 Ab. As a result, an increase in the number of PE-positive cells was confirmed by flow cytometry, and the findings were compared with those obtained with no LPS stimulation (Fig. 5a). PE fluorescence was also observed by fluorescence microscopy using Raw 264.7 cells stimulated with LPS (Fig. 5d, f). In addition, Raw264.7 cells were similarly stimulated and stained with purified rGFP-PDL1scFv. Flow cytometry showed a statistically significant increase in GFP-positive cells (Fig. 5b, c). Furthermore, fluorescence microscopy observed GFP fluorescence in LPS-stimulated Raw264.7 cells (Fig. 5g). In contrast, in the unstimulated cells, only nuclei stained with DAPI were observed (Fig. 5e).

## Discussion

PD-L1 is a well-studied immune checkpoint molecule. Under normal conditions, PD-L1 is primarily expressed by antigen-presenting cells, such as monocytes, macrophages, and dendritic cells. It contributes to host immune homeostasis by binding to PD-1 on T cells, thereby suppressing excessive immunity (Francisco et al. 2010). In contrast, tumor cells are also known to express PD-L1, which enables them to escape antitumor immunity (Ai et al. 2020). The tumor microenvironment accumulates immune cells that highly express PD-L1 and PD-1, and these cell populations have been shown to support tumor survival (Jiang et al. 2019a). Inhibition of PD-1/PD-L1 signaling is a target in developing antitumor strategies. An anti-PD-L1 antibody, atezolizumab, has been developed and approved by the FDA as an antibody-drug (Mathieu et al. 2021). By binding to PD-L1 in the tumor microenvironment, anti-PD-L1 antibodies inhibit the interaction between PD-1 and PD-L1, reducing immunosuppression (Beyrend et al. 2019; Freeman et al. 2000; Iwai et al. 2002). While these effects make anti-PD-L1 antibodies potent antitumor agents, the high cost and labor-intensive nature of producing antibody drugs have been a bottleneck in their adoption (Samaranayake et al. 2009). Against this background, we focused on scFv, a small-molecule antibody, to develop an affordable PD-1/PD-L1-signaling inhibition tool. scFv is a recombinant protein to which a flexible peptide linker attaches to the antigen recognition site of an antibody and which has a binding ability comparable to that of the original antibody (Bird et al. 1988; Ma and O’Kennedy 2017). In addition, because of its simple structure, scFv can be produced in bacterial heterologous protein expression systems. This study employed a gmLAB strain as a host for producing PDL1scFv.

Previously, we reported several gmLAB strains producing scFv and verified their properties, such as their antigen recognition ability (Namai et al. 2020a, b; Shigemori et al. 2017a). Since gmLAB are developed using LAB, they do not contain endotoxin. Therefore, scFv-producing gmLAB can be administered directly without the purification of recombinant protein from the bacterial cell (Shigemori and Shimosato 2017). Oral and intranasal administration of gmLAB has been reported to prevent and/or alleviate disease in animal models by transporting recombinant proteins to local mucosal tissues (Namai et al. 2020c; Yumoto et al. 2020). In this context, we developed a gmLAB strain that produces PDL1scFv as an affordable tool for preventing and alleviating cancer. We consider the findings to be significant because not only is PD-1/PD-L1 signaling prevented using this method, but the presence or absence of PD-L1 expression in the tumor microenvironment is also an important consideration for selecting an appropriate treatment regimen (Shen and Zhao 2018). Consequently, to make the constructed gmLAB better suited for use as a diagnostic/treatment tool, a fusion protein containing GFP added to the scFv was incorporated into the gmLAB.

We first designed the scFv based on the amino acid sequence of atezolizumab, an anti-PD-L1 antibody. The amino acid sequence was obtained by connecting the Fv regions of the heavy and light chains of atezolizumab with a peptide linker, followed by conformational prediction to confirm whether the resulting product was in the form of scFv. The resulting DNA sequence was inserted into the multiple cloning site of the lactococcal nisin-induced GFP expression vector (pNZ8148#2:CYT-GFP) using restriction digestion to create a GFP-fused anti-PD-L1 scFv-producing plasmid (pNZ8148#2:CYT-GFP-PDL1scFv). The resulting vector was introduced into NZ9000 to generate a gmLAB strain, and expression analysis of recombinant scFv was performed by WB. The constructed gmLAB produced rGFP-PDL1scFv only upon adding nisin, a gene expression inducer, confirming that gene expression occurred in a nisin stimulation-dependent manner. The fluorescence of the gmLAB was also examined, and green fluorescence was observed only in nisin-stimulated NZ-GFP and NZ-GFP-PDL1scFv, indicating that GFP produced as a fusion protein exhibits sufficient fluorescence for use as a transformant marker. However, the fluorescence intensity for NZ-GFP-PDL1scFv was weaker than that for NZ-GFP, suggesting that fusion with the scFv reduced expression levels and affected the 3D structure.

Using an ELISA system, we investigated whether rGFP-PDL1scFv exhibits immunoreactivity against PD-L1. Specifically, gmLAB cell lysates that produce rGFP-PDL1scFv were added to the target protein PD-L1-immobilized wells to confirm binding. As a result, the absorbance increased as the concentration of the cell lysate increased. In contrast, no increase in absorbance was observed when PD-L1 was not immobilized. Furthermore, the control gmLAB did not increase absorbance. These results, combined with the fact that the scFv was derived from an α-PD-L1 antibody, suggested that the scFv designed in this study could bind to PD-L1. We verified whether rGFP-PDL1scFv could recognize PD-L1 expressed on the cell surface using cell lines in vitro. Since preclinical studies, such as those using mouse models, will be necessary in the future, experiments were conducted using mouse-derived macrophage cell lines. In addition, atezolizumab, the original antibody for the α-PD-L1 scFv designed in this study, is known to be a humanized human and mouse cross-reactive antibody (Lesniak et al. 2016). Cells from the mouse macrophage cell line, Raw264.7, are known to express PD-L1 protein on the cell surface after stimulation with LPS (Xiao et al. 2020). Here, we describe the binding of rGFP-PDL1scFv to the cell surface PD-L1 using a fluorescence microscope. Furthermore, green fluorescence attributable to GFP was observed when purified rGFP-PDL1scFv was added to LPS-stimulated Raw264.7 cells.

gmLAB based on intracellular production have been reported, and mucosal administration has shown to have desirable effects (Liu et al. 2018). In addition, previous studies have shown that nasally administered gmLAB cleared more than 24 h after administration (Garcia et al. 2018). These results suggest that the administered gmLAB either rupture or are phagocytosed by immune cells and leak intracellular proteins. To verify these processes and to assess the potential further application of the constructed gmLAB, conducting nasal administration experiments using mice will be necessary. In conclusion, we created a gmLAB strain that produces GFP-fused anti-PD-L1 scFv that shows immunoreactivity to PD-L1. Since PD-L1 is a target in many antitumor treatment strategies, and because rGFP-PDL1scFv can recognize PD-L1 and remain fluorescent green, we consider that the gmLAB strain developed in this study has the potential for use as an affordable antitumor agent and diagnostic tool.
